# Digital Epidemiologic Research on Multilevel Risks for HIV Acquisition and Other Health Outcomes Among Transgender Women in Eastern and Southern United States: Protocol for an Online Cohort

**DOI:** 10.2196/29152

**Published:** 2021-04-26

**Authors:** Andrea L Wirtz, Erin E Cooney, Megan Stevenson, Asa Radix, Tonia Poteat, Andrew J Wawrzyniak, Christopher M Cannon, Jason S Schneider, J Sonya Haw, James Case, Keri N Althoff, Elizabeth Humes, Kenneth H Mayer, Chris Beyrer, Allan E Rodriguez, Sari L Reisner, American Cohort To Study HIV Acquisition Among Transgender Women (LITE) Study Group

**Affiliations:** 1 Department of Epidemiology Johns Hopkins Bloomberg School of Public Health Johns Hopkins University Baltimore, MD United States; 2 Callen-Lorde Community Health Center New York, NY United States; 3 Center for Health Equity Research University of North Carolina School of Medicine Chapel Hill, NC United States; 4 Department of Psychiatry and Behavioral Sciences Miller School of Medicine University of Miami Miami, FL United States; 5 Research and Evaluation Whitman-Walker Institute Washington DC, DC United States; 6 Emory University School of Medicine Atlanta, GA United States; 7 School of Nursing Johns Hopkins University Baltimore, MD United States; 8 Beth Israel Deaconess Medical Center Harvard Medical School Harvard University Boston, MA United States; 9 The Fenway Institute Fenway Health Boston, MA United States; 10 Division of Infectious Diseases Department of Medicine University of Miami Miller School of Medicine Miami, FL United States; 11 Division of Endocrinology, Diabetes, and Hypertension Brigham Women’s Hospital Boston, MA United States; 12 Department of Medicine Harvard Medical School Harvard University Boston, MA United States; 13 Department of Epidemiology Harvard TH Chan School of Public Health Harvard University Boston, MA United States

**Keywords:** transgender persons, United States, cohort studies, digital research, HIV infection, HIV testing, public health, online health, transgender, HIV

## Abstract

**Background:**

The HIV epidemic disproportionately impacts transgender women in the United States. Cohort studies identify unique risks for affected populations, but use of facility-based methods may bias findings towards individuals living in research catchment areas, more engaged in health services, or, in the case of transgender populations, those who are open about their transgender identity. Digital clinical trials and other online research methods are increasingly common, providing opportunity to reach those not commonly engaged in research. Simultaneously, there is a need to understand potential biases associated with digital research, how these methods perform, and whether they are accepted across populations.

**Objective:**

This study aims to assess the feasibility of developing and implementing an online cohort of transgender women to assess risks for HIV acquisition and other health experiences. Further, this study aims to evaluate how an online cohort compares to a site-based, technology-enhanced cohort for epidemiologic research. The overarching goal is to estimate incidence of HIV and other health outcomes among transgender women in eastern and southern United States.

**Methods:**

This substudy is part of a larger multisite prospective cohort (LITE) conducted among transgender women, which also includes a site-based, technology-enhanced cohort in 6 eastern and southern US cities. The online cohort was launched to enroll and follow participants across 72 cities in the same region and with similar demographic characteristics as the site-based cohort. Participants are followed for 24 months. Adult transgender women are recruited via convenience sampling (eg, peer referrals, social media, and dating apps). Participants reporting negative or unknown HIV status are enrolled in a baseline study visit, complete a sociobehavioral survey, and provide oral fluid specimens to test for HIV. Participants not living with HIV (lab-confirmed) at baseline are offered enrollment into the cohort; follow-up assessments occur every 6 months.

**Results:**

Enrollment into the online cohort launched in January 2019. Active recruitment stopped in May 2019, and enrollment officially closed in August 2020. A total of 580 participants enrolled into and are followed in the cohort. A recruitment-enrollment cascade was observed across screening, consent, and completion of study activities. Implementation experiences with HIV test kits highlight the need for heavy staff engagement to support participant engagement, visit completion, and retention, even with automated digital procedures.

**Conclusions:**

This study is responsive to increasing research interest in digital observational and intervention research, particularly for populations who are most affected by the HIV epidemic and for those who may otherwise not participate in person. The progression across stages of the recruitment-enrollment cascade provides useful insight for implementation of cohort studies in the online environment.

**International Registered Report Identifier (IRRID):**

DERR1-10.2196/29152

## Introduction

### Background

Transgender women are one of the most affected populations with respect to the HIV epidemic in the United States and are prioritized in the US strategy to end the HIV epidemic [1,2]. Multiple biological, behavioral, and social risks for HIV infection among transgender women are driven by, or are concomitant with, structural barriers that limit access to HIV prevention, testing, care, and health services [3]. Across the United States, transgender women report a high prevalence of sexual assault and violence, homelessness, unemployment, substance use, and low health insurance coverage [4-12]. These factors increase the likelihood of condomless sex, thereby increasing HIV acquisition risk. Due to these multilevel vulnerabilities, transgender women in the United States experience an estimated 14% laboratory-confirmed HIV prevalence (meta-analysis) — more than 42 times the national HIV prevalence in the United States of 0.3% [13]. Significant disparities exist across racial groups and ethnicity: HIV prevalence is estimated to be 44% among Black transgender women and 25% among Latina transgender women [13]. Gender-based discrimination, stigma in health care, and prioritization of basic needs, including gender-affirming care, impede access and uptake of HIV testing, prevention, care, and treatment [12,14,15]. Effective and acceptable HIV prevention interventions tailored to transgender women are urgently needed and require population-specific insight gained from observational and qualitative research [16-21].

HIV prevention research among transgender women faces several challenges. The lack of transgender-specific marketing and misgendering during research (referring to a person by an incorrect name or pronoun) have left a legacy of wariness about HIV research [22]. Inclusion of transgender women as a subset of a larger study population has produced small samples of transgender women, limited scientific inference, and provided insufficient information about acceptable and effective HIV prevention interventions. Despite prior participation in HIV research, transgender women participants have reported that these efforts rarely benefit their communities [22], highlighting the importance of population-specific and community-engaged research to identify and respond to community needs.

In March 2018, we launched the first multisite cohort to assess HIV incidence among transgender women in the United States (known to participants as the LITE study; NIH UG3/UH3AI133669). This cohort has enabled the development and refinement of technology-enhanced methods to reach and retain transgender women in site-based HIV research. The cohort includes 6 technology-enhanced, site-based cohorts in Boston, New York City, Baltimore, Washington DC, Atlanta, and Miami, providing a combination of facility-based in-person and remote assessments for participants over at least 24 months of follow-up [23]. Facility-based visits include self-administered surveys along with HIV and sexually transmitted infection (STI) testing. During remote visits, participants utilize a hybrid app to complete surveys and HIV self-testing, report the results, and upload photos of the test results for validation. This method allowed us to enroll and retain 731 transgender women with varying levels of consistent technology access across these 6 cities.

Cohort studies identify unique risks for affected populations, but the traditional use of facility-based methods may bias findings towards individuals living in research catchment areas, more engaged in health services, or, in the case of transgender populations, those who are open about their transgender identity. Novel, technology-based methods are rapidly increasing in popularity across multiple facets of epidemiologic research. Outside of HIV research, several large cohorts, such as the NIH’s Precision Medicine Initiative Cohort [24] and the Black Women’s Health Study [25], recruit and follow up to 1 million participants across the United States using predominantly technology-based methods. Technology is increasingly integrated into HIV research through sampling and recruitment methods via online advertisements on social media and dating apps as well as electronic peer referral methods [26,27]; electronic and remote data collection [28-30]; and remote specimen collection for biologic measures [31-33]. These tools have permitted enrollment of large samples of participants for behavioral surveys [34-36], cohort research [37-39], and intervention research [40,41]. The development of SMS and smartphone apps has become an increasingly common method to deliver prevention and care interventions [42-45], while gaming methods have been incorporated to maintain participant engagement, particularly among youth, in research activities and to promote health behaviors [45,46]. The potential benefits of improving overall study efficiency, reducing study costs associated with space and staff needs for data collection, and improving convenience for participants suggest that integration of technology into research practices will continue to emerge, expand, and evolve. Yet, counter to these anticipated benefits, there remain questions related to potential biases, additional technology and staff costs, long-term retention, relationship building with communities and participants, participant privacy and security, and feasibility in terms of how these methods perform among diverse populations. Most research efforts focus exclusively on online or site-based cohort methods, limiting the ability to answer these questions.

### Objectives

The objective of the online cohort is to assess the feasibility and qualitative differences in the development and implementation of an online cohort compared to a site-based, technology-enhanced cohort for HIV research. This online cohort will also contribute to the broader aims of the LITE study, which are to (1) determine the efficiency and acceptability of novel, technology-infused recruitment methods to enroll HIV-uninfected transgender women into a prospective cohort; (2) describe the demographic, socioeconomic, behavioral, and physical and mental health profiles of HIV-uninfected transgender women in the eastern and southern United States; (3) estimate HIV incidence among transgender women in high-risk eastern and southern US areas, trends in incidence, and associated individual, social, and structural risk factors; and (4) estimate the HIV Prevention Continuum (HIVPC) among HIV-uninfected participants and the HIV Care Continuum (HIVCC) among transgender women who acquire HIV over the course of follow-up [23]. The overarching goal of these cohorts is to inform subsequent HIV prevention, HIV care, and other health interventions that are developed explicitly to meet the unique health needs of transgender women.

## Methods

### Design

This protocol describes the methods of a “sister” online cohort for transgender women in eastern and southern United States. Online cohort methods are largely developed to match the site-based methods conducted under the LITE study, to the closest extent possible, which has previously been described [23]. The protocol described here provides an overview of the online cohort methods, highlighting distinctions between the online and site-based cohorts. Notably, the online cohort utilizes strictly remote study assessments that include self-administered, electronic surveys and the use of self-collected, oral fluid specimens, which are shipped to a central laboratory for HIV-1 testing. Study assessments occur every 6 months, rather than quarterly visits as in the site-based cohorts. STI testing is not performed due to resource constraints.

All cohort methods have been informed by formative qualitative research, which focused on assessing community perspectives of technology-enhanced and online research methods, HIV testing methods, concerns and suggestions related to HIV research among transgender women, and optimal ways to engage the community [22,47,48].

A virtual community advisory board (CAB) facilitates this research by serving as a mechanism for community consultation for both the site-based and online cohorts and is engaged throughout every phase of the research activities. CAB meetings are held every 4-6 months to discuss research activities, concerns and protection of community members, results, and plans for dissemination to the wider community [49,50].

### Sample

The online cohort enrolled and follows adult transgender women who are not living with HIV in 72 eastern and southern US metropolitan areas with population sizes >100,000. Participating cities for the online cohort were selected on the basis of similar demographic characteristics to the initial 6 cities (Boston, New York City, Baltimore, Washington DC, Atlanta, and Miami) where the site-based cohort is implemented.

Preliminary, self-administered screening assesses for the following inclusion criteria: aged 18 years or older; no prior diagnosis of HIV infection; speaks and understands English or Spanish; and residence in or within a 90-mile radius of one of the following cities: Atlanta, GA; Baltimore, MD; Baton Rouge, LA; Birmingham, AL; Boston, MA; Charlotte, NC; Chicago, IL; Columbia, SC; Detroit, MI; Jackson, MS; Jacksonville, FL; Memphis, TN; Miami, FL; Nashville, TN; New Haven, CT; New Orleans, LA; New York, NY; Newark, NJ; Orlando, FL; Philadelphia, PA; Pittsburgh, PA; Providence, RI; Tampa, FL; Washington, DC; Worcester, MA; Allentown, PA; Aurora, IL; Bridgeport, CT; Buffalo City, NY; Charleston, SC; Charlotte, NC; Chattanooga, TN; Cincinnati, OH; Clarksville, TN; Cleveland, OH; Columbus, GA; Columbus, OH; Fayetteville, NC; Flint, MI; Fort Wayne, IN; Ft. Lauderdale, FL; Hartford, CT; Hollywood, FL; Huntsville, AL; Indianapolis, IN; Jersey City, NJ; Knoxville, TN; Lafayette, LA; Lansing, MI; Lexington, KY; Louisville, KY; Mobile, AL; Montgomery, AL; Norfolk, VA; Palm Bay, FL; Port St. Lucie, FL; Raleigh/Durham, NC; Richmond, VA; Rochester City, NY; Rockford, IL; Savannah, GA; Shreveport, LA; Springfield, NC; Stamford, CT; Syracuse, NY; Tallahassee, FL; Tampa/Clearwater/St. Petersburg, FL; Toledo, OH; Virginia Beach/ Newport News, VA; Waterbury, CT; Wilmington, NC; and Yonkers City, NY. Participants’ transgender status was determined during screening using the recommended two-step method (assigned male sex at birth and current gender identity as a woman or along the trans feminine spectrum) [51,52], inclusive of binary and nonbinary people. Participants who were eligible based on preliminary screening and provided electronic consent to participate were then asked to complete the baseline survey and are sent an oral fluid specimen collection kit for laboratory verification of HIV status and final determination of eligibility in the cohort.

Prior to the consent and enrollment process, participants were asked to complete a 2-factor authentication process (to ensure they are not duplicate enrollees or robotic enrollments). Individuals who are duplicate enrollments or who report concurrent enrollment in an HIV prevention trial were ineligible for participation.

### Recruitment and Enrollment

**Figure 1:**
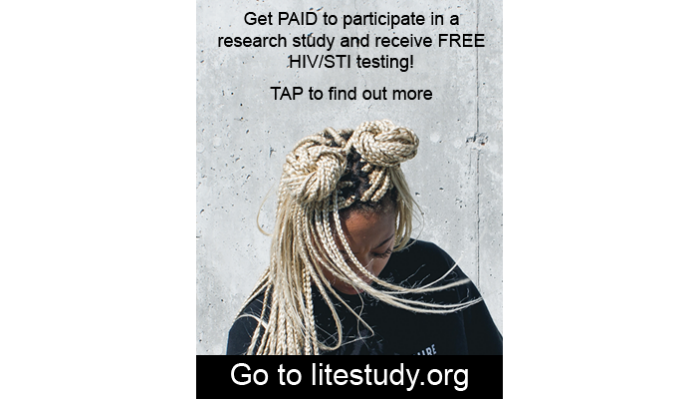
Image of a social media advertisement.

Recruitment utilized a mix of technology-infused methods, including paid advertisements on Google Ads; social media venues frequented by transgender women including Facebook, Instagram, and Reddit; as well as geosocial networking apps used for dating (eg, Grindr and Black Gay Chat; Figure 1). Online advertisements for these sites permitted targeting of ads to the specified cities and surrounding areas, user profiles, or user search terms based on internal algorithms (no individual-level identifying data are shared with researchers). Our study website synchronized with Google Analytics to permit real-time analysis of geographic locations of users, keywords, and sites that referred them, which allowed us to monitor and adjust recruitment strategies as enrollment proceeded.

Advertisements provided a study phone number and link to the study webpage [53]. Participants who followed the link to the webpage could view study information in English or Spanish and access screening and enrollment for the online cohort. Screening assessments were automated with staff review. Individuals living in the 6 site-based cohort cities had the option to enroll in either cohort and could switch cohort at any point over the course of follow-up, depending on their location of residence.

Once a participant provided a unique email on the screening form, was determined to be eligible for the study, and consented to join the study, the system sent a unique link via email. Once this link had been accessed, a verification code was sent via text. On receipt of the text, the participant entered that code into the webpage to complete enrollment. The initial email message that went to consented participants included a link back to this validation page for later use, if a user was not able to provide a phone number at that time. If the code was validated, the user was directed to complete registration and the baseline survey.

Collected identifiers (name, mailing address, telephone number, and email address) are stored separately from the participant survey, and HIV testing data are retained until study end to prevent duplicate enrollments. Individuals using Voice Over Internet Protocol phone numbers are also prevented from enrolling to mitigate fraudulent behaviors or enrollment. An internal algorithm ran real-time checks to identify any duplicate or partially matched identifiers. Identical matches were prohibited from registering; partial matches were reviewed by study staff for final determination.

### Data Collection

Data collection, both survey completion and specimen collection, are supported through the use of a hybrid app developed for this study. The hybrid app enables download of the secure app on Android and iOS operating systems but also provides an optional web-based platform for non-smartphone users. We elected to use a hybrid app, recognizing that while transgender women have high rates of internet use [54], reliance solely on app-based data collection may threaten study retention.

#### Hybrid App

**Figure 2:**
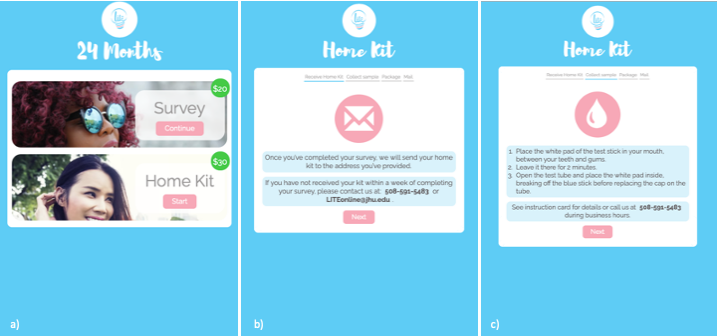
Participant view of the hybrid app of the online cohort, including (A) access to survey or HIV specimen collection kit within a study visit, (B) HIV test home kit shipping confirmation, and (C) home kit specimen collection instructions.

The app, which was developed for the site-based cohorts, was modified for the online cohort to display the participants' study timeline and available activities, change contact information and incentive preferences, complete study surveys, request a self-collection kit for HIV testing, and report when they have sent a specimen to the laboratory (see Figure 2). Participants log into the app using a unique token that is generated at enrollment. Once the app is downloaded, participants can set their own PIN, which is required for subsequent entry to the app. All collected data are encrypted in transit from the device to the secure server, and identifying information is stored encrypted within the database using the AES 256-bit protocol. Staff with role-based access can access a dashboard to view participant study progress, enter lab results, and view contact information for shipment of specimen collection kits to participants. All components of the app and other study materials are available in the English and Spanish languages.

#### Survey Measures

All surveys are self-administered using the study hybrid app. Survey measures span domains set forth by the social ecological model [55] and by the situated vulnerabilities framework [56], including individual, interpersonal, and structural level measures. Unlike the site-based cohort, participants do not complete a literacy screener and do not have an option for staff-administered surveys, given the remote nature of data collection.

Measures have been previously described [23] but briefly include the following: individual-level measures collect data on demographics; access to and use of gender-affirming services [57]; sexual health history and access or uptake of HIV services [58]; health insurance and primary care, mental and behavioral health [59], and barriers and facilitators to care [60-62]; substance use [63-65]; gender affirmation and dysphoria [66]; and engagement in the HIVPC [67]. Interpersonal measures include social support and social network size; detailed partner-by-partner sexual behavior information by partner type and HIV status [68,69]; and measures of intimate and nonpartner physical, sexual, and psychological violence victimization [70]. Structural-level measures include social marginalization and stigma [71]; social capital [72,73]; interactions with the justice system; and measures of immigration status, citizenship, and interactions with immigration detention. As recommended by the CAB, an additional measure of food insecurity was included in the survey [74]. To mitigate risk of incomplete surveys due to survey duration, longer scales that focused on gender pride, community connectedness, and condom use self-efficacy are not included in the online cohort survey.

At the end of the survey, information is provided to the participant about nationally available and LGBTQ-friendly locators for HIV testing, pre-exposure prophylaxis services, and LGBTQ health centers. The survey is also programmed to display locators for nationally available and LGBTQ-friendly mental health services and suicide prevention; violence services; and substance use services for individuals who report mental health symptoms, experiences of violence victimization, or substance use during the course of the survey. Both English and Spanish language resources are included, though Spanish-speaking hotlines often have more limited hours.

#### Biologic Measures

Online cohort participants are asked to provide an oral fluid specimen at baseline and every 6 months for HIV testing. We elected to use oral fluid specimen collection, rather than home HIV self-testing, for 2 reasons: (1) preference to provide confirmatory testing, interactive posttest counseling, and support linkage to care for participants with positive or indeterminate preliminary results and (2) low self-reported response rates of HIV self-test results in other web-based HIV research [75,76]. Participants of the online cohort are not asked to participate in any STI testing or biospecimen storage, unlike site-based cohorts, due to resource constraints and added complexities of shipping specimens to multiple laboratories.

Specimen collection kits are sent by the study staff to the mailing address provided by the study participants. Participants are reminded to provide the name they would like shown on the package; they have the option to provide separate names for mailing vs text and email-based communication. Addresses are validated using the SmartyStreet application programming interface (API). Kits include the oral specimen collection device, OraSure, and easy-to-read bilingual information, including instructions for specimen collection with example photos, prelabeled shipping envelopes with tracking information, and a resource guide with locators for accessing HIV prevention or care resources and culturally appropriate health, mental health, substance use, and violence services.

Participants self-administer the OraSure Oral Specimen Collection Device at home in accordance with the package insert instructions [77]. The device allows for the collection of an oral fluid sample for HIV testing using a pad that is placed between the cheek and gum for 2-5 minutes. The pad is then placed into a sealable vial that contains a preservative, which stabilizes the sample for up to 21 days. Participants mail the vial and any corresponding materials to Quest Diagnostics using the prepaid label that is enclosed in the package. Participants are provided with a 24-hour OraSure Support telephone number as well as the study coordinator number if the participant has questions or difficulty using the collection device.

HIV testing is conducted at Quest Diagnostics Laboratory using an enzyme immune assay, with positive results being confirmed via western blot [78]. Confirmatory testing can take up to 6 additional days to complete. Results of the HIV tests are received by the study team within an average of 7 days. HIV test results are reviewed and provided to the participant within 48-72 hours of receipt via phone for positive results or via their preferred method of contact for negative results. The study staff review the data on a daily basis and call any participant with a positive or indeterminate test to provide posttest counseling and information about how to locate a provider in their location. All participants are informed during the consent process of the requirement to inform local health departments of positive HIV cases, which is performed by Quest Diagnostics per state requirements.

Participants who seroconvert during participation in the online cohort are asked to authorize reviews of their medical records for the purpose of collecting information on their subsequent engagement in HIV care. Participants are informed of their right to decline this request. Cohort (site-based and online) participants who seroconvert during the study will stop participation in the cohort but will be asked to complete one more study visit (in-person or online) at 3 months after the visit in which they tested positive for HIV. This visit serves to assess engagement in the HIVCC among transgender women who are newly diagnosed with HIV.

### Study Retention

We use tracking and retention procedures proven effective in prior studies [23]. Study retention is operationally defined as not missing more than 2 consecutive study assessments. The hybrid app system issues automated text messages and emails to participants’ devices for reminders of follow-up assessments and if study procedures are partially completed. Study staff contact participants by their preferred method (email, phone, text) if participants miss 2 consecutive study visits or regularly complete only part of the study assessment (survey or HIV specimen collection). All participants are provided with a US $50 stipend for the completion of all study activities for each study assessment (US $20 for survey and US $30 for HIV test). We have elected this amount to be consistent with the in-person cohort and other research studies with transgender women in the select cities in conjunction with CAB input. Retention events are held remotely every few months to engage interested participants across the cohorts. Event attendance is optional, and participants can control their level of privacy, such as their displayed name and video. Events have included but are not limited to virtual trivia, film screenings, yoga, and art workshops. Virtual newsletters presenting study updates and preliminary study results across the cohorts are also shared with participants and community members on an annual basis.

### Sample Size

Participants of the online cohort will contribute to the target sample size of 1100 transgender women who are not living with HIV in the LITE study. This target was based on a planned comparison of the incidence of 2 subgroups, assuming a two-sided test statistic and 5% Type 1 error with 80% power. We conservatively estimated 700 person-years of follow-up accumulated after the first 2 years of enrollment and, incorporating a loss of 15% of follow-up time per year, we estimated we will accumulate 935 person-years, 795 person-years, and 676 person-years in years 3, 4, and 5, respectively (>3000 person-years total). With this person-time, we estimated that we would have sufficient power (80%) power to detect public health relevant differences in HIV incidence by various participant characteristics. Relative to other online cohorts [37,39] and as one of the first cohorts among transgender women in the United States, we intentionally kept the sample size small to focus on developing acceptable methods and establishing trust among study participants.

### Analytic Plan

Descriptive analysis will be used to compare differences in basic demographic, behavioral, and health-related characteristics among participants enrolled in the online vs the site-based cohorts, controlling for geographic region of residence. Qualitative differences in recruitment and retention rates will also be observed and described.

Participant data from the online cohort will also contribute to the analysis of overall LITE study, which has been previously described [23]. Briefly, these analyses will include the following: Data visualization techniques will be utilized to compare efficiency of recruitment methods in terms of process measures of time, cost, and response rates, and descriptive analyses will be conducted to compare quantitative measures of acceptability, diversity, and biases of the samples recruited via technology-enhanced and traditional recruitment methods (Aim 1). Descriptive statistics will be conducted to compare demographic and behavioral risk profiles among baseline participants, as well as to assess baseline HIVCC and HIVPC among participants living with and without HIV, respectively (Aim 2). HIV incidence (and 95% confidence intervals) during follow-up will be estimated among the HIV-uninfected cohort (Aim 3). Poisson regression models will be used to investigate important predictors of incident infection. Time-to-event survival models will be used to estimate the risk of HIV seroconversion for the predictors. Finally, the steps in the HIVPC and HIVCC will be investigated over the course of the study using a serial cross-sectional approach to determine trends in these important frameworks (Aim 4). To make these estimates most informative for public health efforts, the steps will be estimated among those under observation in specific calendar years (ie, in 2019, 2020, and 2021).

Where relevant, we will utilize modern missing data techniques [79], with emphasis on multiple-imputation methods, which provide an approach for handling different missing data including coarsely measured [80] or mismeasured [81] variables. Adaptation of these methods to survival analyses will be similar to techniques implemented in R/MICE and SAS/IVEware [82-84]. Statistical analyses will be performed using Stata 14 (StataCorp, College Station TX) and R software (R Foundation for Statistical Computing, Vienna, Austria).

### Human Subjects

Study activities follow a single institutional review board procedure and have undergone review and approval by the Johns Hopkins School of Medicine Institutional Review Board, with reliance by all collaborating organizations and institutions. Protection of participant privacy and confidentiality are central to the development and implementation of all study activities and have been further developed in discussions with the study CAB. The electronic data system has been developed with careful attention to security, including password protection and encrypted data transmission*.*

## Results

**Figure 3:**
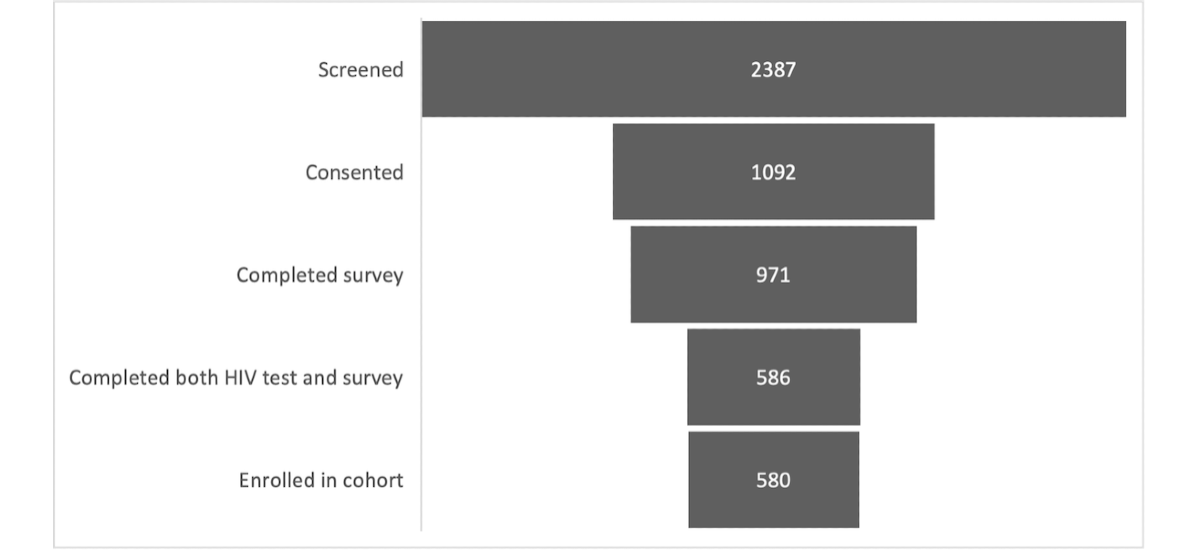
Recruitment-enrollment cascade: progression from screening to enrollment of an online cohort of transgender women.

In January 2019, we launched the online cohort in 25 cities, then expanded in April 2019 to a total of 72 cities in eastern and southern United States. In May 2019, we ended online advertisements, though eligible individuals who had heard of the study from other participants could continue to enroll. Enrollment closed in August 2020, at which point 580 participants were fully enrolled in the online cohort. We observed a recruitment-enrollment cascade in the study implementation process with successive and decreasing rates from online screening through enrollment in the online cohort: 2387 individuals were screened, 1092 individuals consented, 971 individuals completed the survey, 586 individuals completed both the HIV test and survey, and 580 individuals enrolled in the cohort (Figure 3). Ultimately, 45.7% (1092/2387) of those screened were consented, and 53.3% (580/1092) of those consented completed both baseline study activities for full enrollment in the cohort. The self-administered screening form effectively identified ineligible participants on the basis of HIV status, minimizing HIV testing costs, where only 4 participants were newly identified with HIV infection through baseline HIV testing procedures. Discussions with these participants determined that 3 of the 4 were newly diagnosed with HIV, and staff were able to provide appropriate counseling and referral to HIV care for these participants.

**Figure 4:**
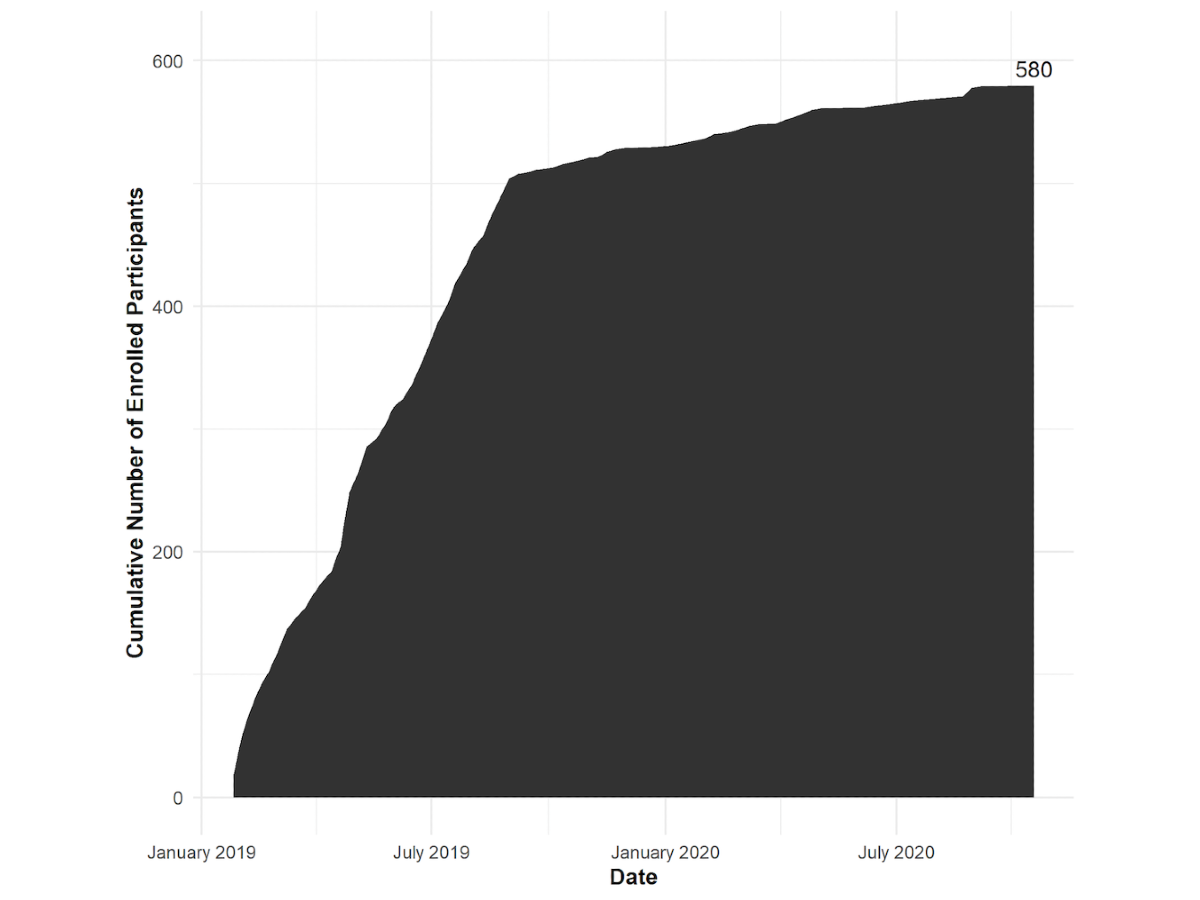
Cumulative number of enrolled participants in the LITE online cohort.

Figure 4 displays cumulative enrollment over time into the online cohort. Over one-third (213/580, 36.8%) of the online sample were aged 18-24 years at enrollment, and one-fifth (125/580, 21.6%) identify as Black, Latinx, or multiracial. Across site-based and online cohorts, we have enrolled and follow 1312 transgender women at risk for HIV in eastern and southern United States. As of the end of October 2020, 573 person-years have been contributed to the analytic dataset of the online cohort. Cohort participants are under follow-up through 2022.

Internal shipping procedures for specimen collection kits have also led to observations of high mobility or unstable housing among cohort participants when specimen collection kits are returned by mail. While requiring more staff effort, returned packages offer an opportunity to identify address changes and connect with participants to support retention. Returned specimen collection kits during enrollment also supported identification of some fraudulent enrollments.

## Discussion

This paper describes the protocol for the development and implementation of an online cohort for assessment of HIV risks and predictors of HIV acquisition among transgender women. In conjunction with the LITE site-based cohort, this study aims to inform priorities and programs of the Ending the HIV Epidemic Strategy for the United States [1,2] and innovates in its use of technology to engage and assess HIV risk among transgender women. Study findings will have important implications for identification of optimal recruitment, retention, and data collection methods for future observational research and digital or hybridized digital clinical trials among transgender women living with and without HIV infection, estimation of HIV incidence, and ultimately the development of acceptable HIV prevention strategies for ending the HIV epidemic among transgender women in the United States. As with other online cohorts and cross-sectional studies [37,39,75], we noted a recruitment-enrollment cascade from screening through enrollment and completion of study activities, which has important design, budgetary, and logistical implications for future digital epidemiologic studies and clinical trials.

This online cohort is strengthened by extensive formative research and early experiences incorporating technology-enhanced methods into the site-based cohorts [22,23,47,48]. Though we make every effort to ensure that technology-enhanced and digital methods are accessible, there is a risk that individuals with extremely limited access to technology may be excluded from this online cohort. Thus, the rare ability to compare study findings across site-based and online cohorts provides invaluable information about sources of bias, but more importantly indicates if use of digital methods risks excluding subgroups who could most benefit from HIV research and subsequent HIV prevention and care interventions.

## Abbreviations

API: application programming interface
CAB: community advisory board
HIVCC: HIV Care Continuum
HIVPC: HIV Prevention Continuum
JHU: Johns Hopkins University
PI: principal investigator
STI: sexually transmitted infection
